# Submaximal exercise blood pressure and cardiovascular structure in adolescence^☆^

**DOI:** 10.1016/j.ijcard.2018.10.060

**Published:** 2019-01-15

**Authors:** Martin G. Schultz, Chloe Park, Abigail Fraser, Laura D. Howe, Siana Jones, Alicja Rapala, George Davey Smith, James E. Sharman, Deborah A. Lawlor, Nish Chaturvedi, John Deanfield, Alun D. Hughes

**Affiliations:** a Menzies Institute for Medical Research, University of Tasmania, Hobart, Australia; b Institute of Cardiovascular Sciences, University College London, London, UK; c MRC Integrative Epidemiology Unit, University of Bristol, Bristol, UK; d School of Social and Community Medicine, University of Bristol, Bristol, UK; e NIHR Biomedical Research Centre, University Hospitals Bristol NHS Foundation Trust and the University of Bristol, UK

**Keywords:** ALSPAC, Hypertension, Exercise, Echocardiography, Body composition, Adolescent

## Abstract

**Purpose:**

Dynamic exercise results in increased systolic blood pressure (BP). Irrespective of resting BP, some individuals may experience exaggerated rise in systolic BP with exercise, which in adulthood is associated with risk of hypertension, and cardiovascular (CV) disease. It is unknown if exercise BP is associated with markers of CV structure during adolescence. We examined this question in a large adolescent cohort taking account of the possible confounding effect of body composition and BP status.

**Methods:**

4036 adolescents (mean age 17.8 ± 0.4 years, 45% male), part of a UK population-based birth cohort study completed a sub-maximal step-test with BP immediately post-exercise. Sub-samples underwent comprehensive echocardiography for assessment of cardiac structure; arterial structure including aortic pulse wave velocity (PWV) and carotid intima-media thickness; and assessment of body composition by dual-energy X-ray absorptiometry (DXA).

**Results:**

Each 5 mm Hg higher post-exercise systolic BP was associated with CV structure, including 0.38 g/m^2.7^ (95% CI: 0.29, 0.47) greater left-ventricular mass index (LVMI), and 0.04 m/s (95% CI: 0.03, 0.04) greater aortic PWV. Adjustment for age, total body fat, lean mass and BP status attenuated, but did not abolish associations with LVMI (0.14 g/m^2.7^ per 5 mm Hg of post-exercise systolic BP; 95% CI 0.21, 0.39) or aortic PWV (0.03 m/s per 5 mm Hg of post-exercise systolic BP; 95% CI: 0.02, 0.04).

**Conclusion:**

Submaximal exercise systolic BP is associated with markers of CV structure in adolescents. Given the clinical relevance of exercise BP in adulthood, such associations may have implications for CV disease screening in young people and risk in later life.

## Highlights

• The blood pressure response to a simple submaximal exercise test was associated with cardiovascular structure in adolescents.
• Exercise blood pressure and cardiovascular structure associations are independent of body composition and blood pressure status.
• Submaximal exercise testing with blood pressure measurement may be a useful cardiovascular disease screening tool in adolescents.

## 1 Introduction

Dynamic incremental exercise increases the metabolic needs of active musculature, resulting in a normal stepwise elevation to systolic BP with increasing exercise intensity. In adulthood, excessive elevation in systolic BP with acute exercise, ‘exaggerated exercise blood pressure (EEBP)’ is associated with heightened levels of cardiovascular disease (CVD) risk, independent of resting BP. Clinical studies indicate that an EEBP response to submaximal exercise testing predicts incident CVD [1], hypertension [2], and reveals underlying high BP otherwise un-detectable via traditional (resting) screening methods [3,4]. Moreover, in adults EEBP is associated with markers of sub-clinical CVD, including raised left-ventricular (LV) mass, altered cardiac function/mechanics and increased arterial stiffness [[5], [6], [7]]. Whilst these data demonstrate the potential clinical importance of EEBP as an established CVD risk marker in adulthood, little is known about exercise BP and whether it is associated with cardiovascular structure during earlier life or adolescence. Indeed, associations between exercise BP and cardiovascular structure in adolescence, whether physiological or pathological in nature, may provide some insight into potential for future CVD. The aim of this study was to determine if submaximal exercise BP was associated with measures of cardiac and arterial structure in a cross-sectional analysis of males and females drawn from a large UK population-based cohort of adolescents. Since body composition explains a large amount of the variance in adolescent cardiac structure, we also sought to examine the potential influence of body composition (total fat and lean body mass) assessed by dual-energy X-ray absorptiometry (DEXA) on associations between exercise BP and cardiovascular structure.

## 2 Methods

### 2.1 Participants

Data were from the Avon Longitudinal Study of Parents and Children (ALSPAC), a large ongoing UK prospective birth cohort study. Details of the ALSPAC design, cohort and timing of examinations have been previously reported [8]. The total sample size for analyses using any data collected after the age of seven is 15,247 pregnancies, resulting in 15,458 fetuses. Of this total sample, 14,775 were live births and 14,701 were alive at 1 year of age. Please note that the study website (http://www.bris.ac.uk/alspac/researchers/data-access/data-dictionary) contains details of all the data that is available through a fully searchable data dictionary. For this cross-sectional analysis, eligible participants included 4036 individuals (45% male) who completed a sub-maximal exercise step test with BP measurement immediately post-exercise at the ALSPAC 17-year follow-up (mean participant age 17.8 ± 0.4 years). This was the only wave of ALSPAC in which a sub-sample of individuals also had detailed cardiovascular assessments including cardiac echocardiography (*n* = 2102), carotid-femoral (aortic) pulse wave velocity (PWV) measurements (*n* = 3582), and carotid intima-media thickness (IMT) measures (*n* = 3746). Individuals with a known history of CV disease (*n* = 31), type one diabetes using insulin therapy (*n* = 14) or who were pregnant (*n* = 8) were excluded from this analysis. Ethical clearance was granted by the ALSPAC Law and Ethics Committee and the Local Research Ethics Committee and all participants provided written and informed consent.

### 2.2 Exercise test and blood pressure measurement

Participants undertook a modified Tecumseh Step Test. This submaximal exercise test involved stepping onto and off a step (a standardized 20 cm high) using both feet for 3 min at a fixed cadence of 23 steps per minute. A metronome was used to set the tempo of steps. The test was terminated upon completion or on request of the participant. Heart rate was recorded immediately prior to the exercise test, immediately post the exercise test and at 3 min in recovery from the exercise test. A crude estimate of intensity of effort achieved during the test was calculated as post-exercise heart rate as a percentage of theoretical maximum heart rate (220-age). A validated Omron 705 IT (Omron Electronic Components Europe BV) BP monitor [9], with appropriate sized cuff was used to measure systolic and diastolic BP and heart rate. A single BP was recorded immediately prior to the test with the participant standing, arm relaxed at side (pre-exercise BP). The participant then continued to wear the BP cuff during the step-test with a further single BP measure recorded immediately upon test cessation (within the first 30 s; post-exercise BP), and again following a 3-minute recovery period (recovery-exercise BP). Mean arterial pressure (MAP) was derived from the formula ((0.33 ∗ (systolic BP − diastolic BP)) + diastolic BP). Rate-pressure product (RPP) was calculated as the product of systolic BP and pulse rate at the time of post-exercise measurements. Office BP (resting) was also recorded as the average of the final 2 of 3 seated measures using the same device [10], at a time separate from the exercise test, but on the same day. Office BP was classified as raised on the basis of pediatric hypertension thresholds as systolic and/or diastolic BP ≥95th percentile cut-points for gender and height at age 17 [11], and alternatively on the basis of the adult hypertension threshold as systolic and/or diastolic BP ≥140/90 mm Hg [10].

### 2.3 Cardiac structure

Echocardiography was performed using HDI 5000 (Phillips Healthcare, North Andover, Massachusetts, USA) ultrasound with integrated P4-2 phased array ultrasound transducer. All cardiac structural measurements (each taken at end-diastole) and analyses were made according to American Society of Echocardiography (ASE) guidelines [12]. Two trained cardiac sonographers measured and analyzed all variables. In a subsample (*n* = 30), intra-class correlation coefficients (ICC) calculated for observations within (ICC range 0.83–0.93) and between (ICC range 0.85–0.92) sonographers were considered excellent. LV geometry was defined on the basis of cut-offs of LV mass index and relative wall thickness (RWT) provided by Lang et al. [12] Therefore, concentric remodeling was defined as LV mass index <35.01 g/m^2.7^ (females) or < 38.30 g/m^2.7^ (males) and RWT >0.46. Concentric hypertrophy was defined as LV mass index ≥35.01 g/m^2.7^ (females) or ≥38.30 g/m^2.7^ (males) and RWT >0.46. Eccentric hypertrophy was defined as LV mass index ≥35.01 g/m^2.7^ (females) or ≥38.30 g/m^2.7^ (males) and RWT <0.46.

### 2.4 Vascular structure

A measure of regional artery stiffness was calculated by ECG-gated carotid-femoral (aortic) pulse wave velocity (PWV) using the Vicorder device (Version 5.1; Skidmore Medical Ltd) and following consensus guidelines [13]. Assessment of carotid intima-media thickness (cIMT) was undertaken on the right and left common carotid arteries via high-resolution B ultrasound, imaged longitudinally 1–2 cm proximal to the carotid bifurcation. Off-line quantitative analysis was undertaken by several independent technicians using a semi-automatic analysis program (Vascular Research Tools 5, Medical Imaging Applications, LLC 2008). Left and right measurements were averaged and reported at end-diastole.

### 2.5 Body composition and blood biochemistry

Height was measured unshod to nearest 0.1 cm using a Harpenden Stadiometer and weight was measured in light clothing to the nearest 0.1 kg using a Tanita TBF 305 scales. Body mass index was calculated as weight (in kg)/height (in meters)^2^. Estimates of total fat and lean body mass were made by DEXA scanner (Lunar Prodigy DXA scanner; GE Medical Systems, Madison, WI, USA). Blood draw following overnight fasting or a minimum of 6 h for afternoon/evening appointments, and biochemistry analysis of glucose and cholesterol including triglycerides, HDL, and LDL (not directly measured but calculated from total, HDL and triglycerides) cholesterol was undertaken following locally established procedures.

### 2.6 Statistical analysis

All analyses were conducted using IBM SPSS statistics (version 22). Continuous data are summarized as mean (SD), categorical data as *n* (%). Sex differences in continuous variables were assessed by *t*-tests, and categorical variables by chi-square analyses. Multivariable linear regression models were constructed using CVD structural measures with known prognostic value in adults as outcomes (i.e. LV mass, RWT, PWV, cIMT) and exercise test BPs (pre-exercise, post-exercise and recovery-exercise systolic BP) as the primary independent variables in each model. Results were presented as β coefficients (95% confidence interval) per 5 mm Hg increases in systolic BP. Sex-combined models were constructed for each outcome, unless there was a significant sex*post-exercise or sex*recovery-exercise systolic BP interaction in associations with the outcome of interest, in which case sex specific models were constructed. Multiple imputation (using the iterative MCMC method, 10 iterations, pooling average of 5 imputed data-sets) was also performed to account for missing data in multivariable models. As the extent of missing data was minimal (<10% for each variable of interest), and since this didn't substantially alter associations, only the results of the complete-case analyses have been presented. Normality of all variables was examined through visualization of distributions and Q-Q distribution plots. Assumptions for linear regression were assessed by inspection of residuals and a tolerance level < 0.10 was interpreted as indicating collinearity.

## 3 Results

### 3.1 Participant characteristics

Male participants had lower total cholesterol, HDL and LDL cholesterol, but higher fasting glucose levels compared to females. There were few smokers in the cohort and there appeared to be little difference between males and females. <1% of the study population had self-reported physician diagnosed hypertension, although raised office BP occurred more commonly in males compared to females, irrespective of adolescent or adult hypertension definition (Table 1a). Whilst on average female participants were shorter and weighed less than male counterparts, BMI and DEXA measured total body fat mass and body fat percentage were greater. Males had greater total body lean mass than females (Table 1b).

**Table 1:** Demographic and clinical characteristics, body composition and exercise test parameters.

	Mean ± SD or N (%) in all participants	Mean ± SD or N (%) in females	Mean ± SD or N (%) in males	*p* value
a. Demographic/clinical				
Age, years (*n* = 4036)	17.8 ± 0.4	17.8 ± 0.4	17.8 ± 0.4	0.797
Cholesterol, mmol/l (*n* = 2673)	3.75 ± 0.67	3.9 ± 0.7	3.6 ± 0.6	<0.001
Triglycerides, mmol/l (*n* = 2673)	0.83 ± 0.36	0.83 ± 0.34	0.83 ± 0.38	0.974
HDL, mmol/l (*n* = 2673)	1.27 ± 0.30	1.35 ± 0.32	1.18 ± 0.26	<0.001
LDL, mmol/l (*n* = 2673)	2.10 ± 0.60	2.20 ± 0.62	1.99 ± 0.56	<0.001
Fasting glucose, mmol/l (*n* = 2673)	5.02 ± 0.40	4.91 ± 0.36	5.14 ± 0.41	<0.001
Hypertension diagnosis (*n* = 4036)	28 (0.7)	18 (0.8)	10 (0.6)	0.320
Current smoker^a^ (*n* = 4036)	414 (10.3)	237 (10.7)	177 (9.8)	0.293
Office BP raised adolescent^b^ (*n* = 3942)	28 (7.5)	117 (5.4)	179 (10.1)	0.320
Office BP raised adult^c^ (*n* = 3918)	118 (3.0)	14 (0.6)	104 (5.7)	<0.001
b. Body composition				
Height, m (*n* = 3942)	1.71 ± 0.09	1.65 ± 0.06	1.79 ± 0.07	<0.001
Weight, kg (*n* = 3946)	66.8 ± 13.2	62.5 ± 11.9	72.2 ± 12.8	<0.001
Body mass index (*n* = 3942)	22.7 ± 3.9	22.9 ± 4.0	22.6 ± 3.7	0.015
Body fat, % (*n* = 3907)	25.4 ± 11.5	33.4 ± 7.0	15.6 ± 7.7	<0.001
Total fat mass, kg (*n* = 3875)	17.9 ± 10.1	21.4 ± 9.1	13.8 ± 9.6	<0.001
Total lean mass, kg (*n* = 3875)	45.7 ± 10.0	38.0 ± 4.3	55.2 ± 6.2	<0.001
c. Exercise test parameters				
Pre-exercise SBP, mm Hg (*n* = 3984)	121 ± 13	117 ± 11	127 ± 13	<0.001
Pre-exercise DBP, mm Hg (*n* = 3984)	74 ± 9	74 ± 9	73 ± 9	0.376
Pre-exercise MAP, mm Hg (*n* = 3984)	89 ± 9	88 ± 9	91 ± 9	<0.001
Pre-exercise heart rate, bpm (*n* = 3983)	82 ± 13	84 ± 13	79 ± 13	<0.001
Post-exercise SBP, mm Hg (*n* = 4036)	143 ± 16	141 ± 16	145 ± 17	<0.001
Post-exercise DBP, mm Hg (*n* = 4036)	80 ± 10	81 ± 11	79 ± 10	<0.001
Post-exercise MAP, mm Hg (*n* = 4036)	101 ± 10	101 ± 10	102 ± 10	<0.001
Post-exercise heart rate, bpm (*n* = 4036)	105 ± 21	112 ± 21	96 ± 18	<0.001
Post-exercise RPP (*n* = 4036)	14,886 ± 3426	15,716 ± 3526	13,865 ± 3000	<0.001
Recovery-exercise SBP, mm Hg (*n* = 3951)	128 ± 13	125 ± 12	132 ± 13	<0.001
Recovery-exercise DBP, mm Hg (*n* = 3951)	81 ± 9	81 ± 9	81 ± 9	0.365
Recovery-exercise MAP, mm Hg (*n* = 3951)	96 ± 9	95 ± 97	97 ± 9	<0.001
Recovery exercise heart rate, bpm (*n* = 3951)	88 ± 15	91 ± 15	85 ± 14	<0.001
Percentage of max heart rate achieved, %	52 ± 11	55 ± 10	47 ± 9	<0.001
Change heart rate pre- to post-exercise, bpm	23 ± 17	28 ± 17	17 ± 13	<0.001

Data are mean ± SD or *n* (%). HDL, high-density lipoprotein cholesterol; LDL, low-density lipoprotein cholesterol; SBP, systolic blood pressure; DBP, diastolic blood pressure; RPP, rate-pressure product. *p* values relate to comparison of males vs. females and were calculated using a *t*-test or Chi^2^ test as appropriate.

a Defined as currently smoking at least one cigarette every day.

b Systolic and/or diastolic BP ≥95th percentile cut-points for gender, and height at age 17.

c Systolic and/or diastolic BP ≥140/90 mm Hg.

### 3.2 Exercise test parameters

Males had greater systolic BP, with heart rate measures at pre-exercise, post-exercise and in recovery-exercise compared with female participants. Diastolic BP did not differ by sex at pre-exercise or in recovery, but was lower post-exercise in males. MAP was marginally higher in males' pre-exercise, post-exercise and in recovery-exercise compared to females. Post-exercise RPP was lower in males compared with females (Table 1c). 94% of participants had an increase in systolic BP (≥1 mm Hg) from pre-exercise to post-exercise, with the mean change being higher for females when compared to males (24 ± 13 mm Hg vs.18 ± 13 mm Hg, *p* < 0.001). Females appeared to achieve a higher percentage of maximum heart rate compared to males. Post-exercise systolic BP was associated with both total fat and lean body mass (*r* = 0.193 and 0.176), and height, weight and BMI (*r* = 0.114, 0.296 and 0.271) in sex-pooled analysis.

**Fig. 1:**
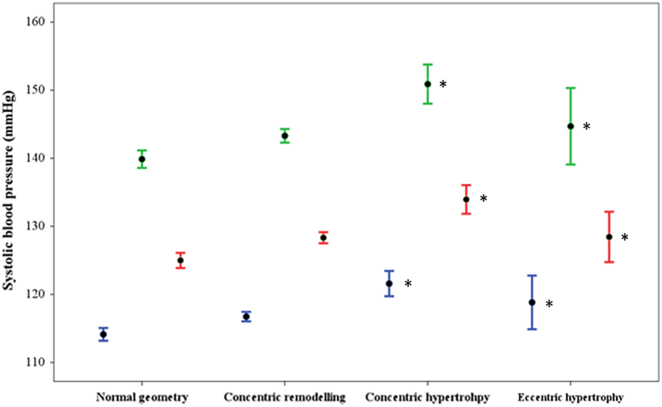
Office resting (blue bars), post-exercise (green bars) and recovery-exercise (red bars) systolic blood pressure (BP) according to left ventricular (LV) geometric pattern. Systolic BPs were highest in those with concentric hypertrophy by comparison to those with normal LV geometry. Solid markers indicate the mean systolic BP. Error bars indicate 95% confidence intervals of the mean values. *p < 0.05 vs. normal geometry.

**Table 2:** Pre-exercise, post-exercise and recovery-exercise systolic BP and cardiovascular structure (sex-pooled analyses).

	Model 1	Model 2	Model 3	Model 4	Model 5	Model 6	Model 7
	β (95% CI) per 5 mm Hg of systolic BP	β (95% CI) per 5 mm Hg of systolic BP	β (95% CI) per 5 mm Hg of systolic BP	β (95% CI) per 5 mm Hg of systolic BP	β (95% CI) per 5 mm Hg of systolic BP	β (95% CI) per 5 mm Hg of systolic BP	β (95% CI) per 5 mm Hg of systolic BP
A. Pre-exercise systolic BP							
LV Mass, g (*n* = 1768)	4.883 (4.359, 5.407)^a^	4.884 (4.359, 5.408)^a^	4.857 (4.335, 5.378)^a^	1.253 (0.816, 1.690)^a^	0.729 (0.324, 1.133)^a^	0.617 (0.173, 1.061)^a^	0.541 (−0.022, 1.105)
LVMI, g/m^2.7^ (*n* = 1756)	0.568 (0.464, 0.671)^a^	0.568 (0.464, 0.671)^a^	0.555 (0.456, 0.655)^a^	0.339 (0.226, 0.452)^a^	0.216 (0.110, 0.323)^a^	0.191 (0.076, 0.307)^a^	0.145 (−0.002, 0.293)
LA size, cm (*n* = 1603)	0.038 (0.031, 0.045)^a^	0.038 (0.031, 0.045)^a^	0.038 (0.031, 0.045)^a^	0.012 (0.005, 0.020)^a^	0.004 (−0.003, 0.011)	0.000 (−0.002, 0.013)	0.010 (0.000, 0.019)
RWT (*n* = 1768)	0.002 (0.001, 0.003)^a^	0.002 (0.001, 0.003)^a^	0.002 (0.001, 0.003)^a^	0.002 (0.001, 0.003)^a^	0.002 (0.001, 0.003)^a^	0.002 (0.001, 0.003)^a^	0.001 (−0.001, 0.002)
Aortic PWV, m/s (*n* = 2965)	0.068 (0.060, 0.077)^a^	0.068 (0.059, 0.077)^a^	0.069 (0.060, 0.078)^a^	0.041 (0.032, 0.050)^a^	0.043 (0.033, 0.052)^a^	0.037 (0.030, 0.045)^a^	0.015 (0.002, 0.027)^a^
cIMT, mm (*n* = 3687)	0.002 (0.001, 0.002)^a^	0.002 (0.001, 0.002)^a^	0.002 (0.001, 0.002)^a^	0.001 (0.000, 0.001)^a^	0.001 (0.000, 0.001)^a^	0.001 (0.000, 0.002)^a^	0.000 (0.000, 0.001)
B. Post-exercise systolic BP							
LV Mass, g (*n* = 1747)	2.357 (1.908, 2.806)^a^	2.357 (1.908, 2.807)^a^	2.239 (1.784, 2.695)^a^	1.094 (0.771, 1.417)^a^	0.481 (0.174, 0.789)^a^	0.411 (0.096, 0.725)^a^	0.301 (−0.067, 0.668)
LVMI, g/m^2.7^ (*n* = 1734)	0.383 (0.298, 0.468)^a^	0.383 (0.299, 0.468)^a^	0.302 (0.219, 0.385)^a^	0.295 (0.212, 1.379)^a^	0.152 (0.071, 0.232)^a^	0.131 (0.049, 0.213)^a^	0.092 (−0.003, 0.188)
LA size, cm (*n* = 1580)	0.023 (0.017, 0.029)^a^	0.023 (0.017, 0.029)^a^	0.018 (0.012, 0.024)^a^	0.014 (0.008, 0.019)^a^	0.004 (−0.002, 0.009)	0.004 (−0.001, 0.009)	0.005 (−0.001, 0.011)
RWT (*n* = 1747)	0.002 (0.001, 0.002)^a^	0.002 (0.001, 0.002)^a^	0.002 (0.001, 0.002)^a^	0.002 (0.001, 0.002)^a^	0.001 (0.001, 0.002)^a^	0.001 (0.000, 0.002)	0.001 (0.000, 0.002)
Aortic PWV, m/s (*n* = 2902)	0.038 (0.030, 0.045)^a^	0.037 (0.030, 0.045)^a^	0.044 (0.036, 0.051)^a^	0.026 (0.019, 0.033)^a^	0.029 (0.022, 0.037)^a^	0.029 (0.021, 0.037)^a^	0.010 (0.001, 0.019)^a^
cIMT, mm (*n* = 3607)	0.001 (0.000, 0.001)^a^	0.001 (0.000, 0.001)	0.001 (0.001, 0.001)^a^	0.000 (0.000, 0.001)	0.000 (0.000, 0.001)	0.001 (0.000, 0.001)	0.000 (0.000, 0.001)
C. Recovery-exercise systolic BP							
LV Mass, g (*n* = 1708)	3.984 (3.439, 4.530)^a^	3.986 (3.440, 4.531)^a^	3.872 (3.325, 4.420)^a^	1.246 (0.826, 1.666)^a^	0.513 (0.119, 0.908)^a^	0.417 (0.010, 0.825)^a^	0.226 (−0.266, 0.718)
LVMI, g/m^2.7^ (*n* = 1695)	0.534 (0.430, 0.639)^a^	0.381 (0.295, 0.466)^a^	0.466 (0.365, 0.568)^a^	0.356 (0.248, 0.464)^a^	0.185 (0.082, 0.287)^a^	0.159 (0.053, 0.265)^a^	0.111 (−0.017, 0.239)
LA size, cm (*n* = 1543)	0.032 (0.025, 0.040)^a^	0.032 (0.025, 0.039)^a^	0.027 (0.020, 0.035)^a^	0.013 (0.006, 0.020)^a^	0.001 (−0.006, 0.008)	0.001 (−0.006, 0.008)	0.001 (−0.008, 0.010)
RWT (*n* = 1708)	0.002 (0.002, 0.003)^a^	0.003 (0.002, 0.004)^a^	0.003 (0.002, 0.003)^a^	0.002 (0.001, 0.003)^a^	0.002 (0.001, 0.003)^a^	0.002 (0.001, 0.003)^a^	0.001 (0.000, 0.003)
Aortic PWV, m/s (*n* = 2846)	0.068 (0.058, 0.077)^a^	0.068 (0.058, 0.077)^a^	0.071 (0.062, 0.081)^a^	0.043 (0.033, 0.052)^a^	0.045 (0.036, 0.055)^a^	0.047 (0.037, 0.057)^a^	0.024 (0.011, 0.036)^a^
cIMT, mm (*n* = 3531)	0.002 (0.001, 0.002)^a^	0.002 (0.001, 0.002)^a^	0.002 (0.001, 0.002)^a^	0.001 (0.000, 0.001)	0.001 (0.000, 0.001)	0.001 (0.000, 0.001)	0.000 (0.000, 0.001)

Results presented as unit change (β) in outcome per 5 mm Hg increase in systolic BP. LV, left-ventricular; LA, left-atrial; RWT, relative wall thickness; cIMT, carotid intima media thickness; PWV, pulse wave velocity. **Model 1** - univariable; **Model 2** - adjusted for age (years); **Model 3** - model 2 plus adjustment for total body fat mass (kg); **Model 4** - model 2 plus adjustment for lean mass (kg); **Model 5** - model 2 plus adjustment for total body fat mass (kg) and total lean mass (kg); **Model 6** - model 5 plus adjustment for hypertension status (resting SBP and/or DBP ≥ 140/90 mm Hg - yes/no); **Model 7** - model 5 plus adjustment for office resting SBP.

a Indicates confidence intervals do not cross zero.

### 3.3 Cardiovascular structure and exercise test systolic BP

**Supplementary Fig. 1:**
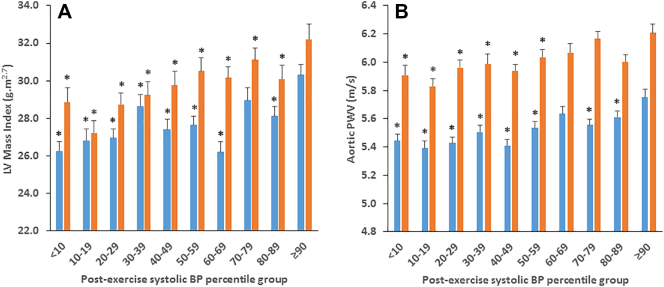
Trends for increased LV mass index (panel A) and aortic PWV (panel B) across post-exercise systolic BP percentile groups for both males (orange) and females (blue). * indicated different values of LV mass index and aortic PWV compared with the ≥90th percentile group of post-exercise systolic BP.

Males had higher values for all cardiac structural variables, as well as greater aortic PWV and carotid IMT compared to females, although effect sizes were only small to moderate (Supplementary Table 1). Males and females with post-exercise systolic BP ≥90th percentile demonstrated greater LV mass index compared with individuals in all other percentile groups, excluding the 70–79th percentile group in females (Supplementary Fig. 1a). Males and females with post-exercise systolic BP ≥90th percentile demonstrated greater aortic PWV compared to individuals in each percentile group below the 60th percentile (Supplementary Fig. 1b). Whilst majority of participants had normal LV geometry, 1.9% (*n* = 40) had indication of concentric hypertrophy and 8.0% (*n* = 167) eccentric hypertrophy. Pre-exercise, post-exercise and recovery-exercise systolic BP were all higher in those with concentric and eccentric hypertrophy compared to those with normal LV geometry (Fig. 1). There were however no differences in the change in systolic BP (delta) from pre-exercise to post-exercise between any of the four remodeling categories, nor percentage of heart rate maximum achieved during the exercise step test. Those with eccentric hypertrophy had greater total body lean mass compared to those with normal LV geometry (48.7, 95% CI 47.2–50.4 vs. 45.3, 44.8–45.8 kg), similar total body fat mass to those with concentric hypertrophy, but greater than those with normal LV geometry (26.4, 24.7–28.0 and 26.0, 22.5–29.4 vs. 17.4, 16.9–17.9 kg respectively).

### 3.4 Exercise test BP and associations with cardiovascular structure

Since there was an absence of sex*exercise systolic BP interactions on any outcome of interest, sex-pooled multiple regression analysis was conducted. Each 5 mm Hg increase in pre-exercise, post-exercise and recovery-exercise systolic BP was associated with increases to LV mass, LVMI, LA size, RWT, aortic PWV and carotid IMT (model 1, Table 2). All associations remained similar with adjustment for age (model 2, Table 2). The addition of DEXA measured total body fat mass (model 3, Table 2) marginally attenuated the degree of association with all outcomes. Replacing fat mass with total body lean mass (model 4, Table 2) for most outcomes, attenuated the strength of associations. Model 5, Table 2 included both total body fat and lean mass, and this attenuated all associations. Including hypertension status in model 6 (irrespective of adolescent or adult definition; adult definition shown) further attenuated the strength of relationships between pre-exercise, post-exercise and recovery-exercise systolic BP and each outcome variable. Replacing hypertension status with clinic measured resting systolic BP (model 7) attenuated all associations. An additional model that included glucose, HDL and LDL cholesterol as additional covariates to those presented in model 6 was constructed. Regression models with delta systolic BP (change in systolic BP from pre-exercise to post-exercise) as the primary independent variable are displayed in Supplementary Table 2, and broadly indicate no association with cardiac structure in all adjusted models.

## 4 Discussion

In this large group of adolescents, post-exercise and recovery-exercise systolic BP was associated with CV structure independent of body composition and BP status. Since in adulthood, an EEBP recorded during, or immediately post-submaximal exercise, is associated with CVD outcomes (including CVD events and mortality), these findings may have important ramifications for BP-related risk screening in adolescents, and CVD health in later life.

Recent synthesis of longitudinal data has highlighted the potential clinical value of an EEBP response to clinical exercise testing for predicting future hypertension, CVD events and mortality [1,2]. Whilst not fully understood, mechanisms underlying the risk associated with EEBP are likely multifactorial. The principal signs of hypertension-related organ damage include structural adaption to the heart (increased LV mass and wall thickness) and large arteries (i.e. arteriosclerosis); these may be a consequence of pathological remodeling resulting from repeated cyclic stress on the CV system. Chronically raised arterial BP alters structural properties of the large conduit arteries [14], and thus increases work required by the left ventricle to eject blood into the system. Average values of CV structure were within the normal range in this apparently healthy adolescent cohort, and few participants exhibited indication of concentric hypertrophy. However, in the setting of acute exercise, the requirement for elevated cardiac output, perhaps ejected into an already stiffened and less compliant arterial system would theoretically result in a greater exercise systolic BP response, and underlie the observed associations with CV structure. On the other hand systolic BP (pre-exercise, post-exercise and recovery-exercise) was also higher in those with eccentric LV hypertrophy compared to those with normal LV geometry, and thus observed associations between systolic BP and cardiac structure could be explained by physiological, rather than pathological adaptations. This is perhaps, less likely, since physiological adaptations to the left ventricle typically do not occur in the absence of a substantial endurance exercise training load [15], and we observed no apparent differences in an index of fitness (percentage maximum heart rate achieved) in relation to delta systolic BP from pre-exercise to post-exercise across the four LV structural remodeling categories.

In selected adult populations (including apparently healthy individuals and those with prehypertension and/or diabetes), markers of CV dysfunction, including impaired endothelial vasodilator function [7], dyslipidemia [16], carotid atherosclerosis [17], and insulin resistance assessed by homeostasis model of insulin resistance (HOMR-IR) [18], have been associated with an EEBP. Data from cross-sectional studies also show EEBP to be related to end-organ damage (including raised LV mass) [5], and sub-clinical disease markers such as large artery stiffness [16,19]. Whilst an EEBP is relatively common in adults with established CVD risk factors or clinical conditions (e.g. those with type 2 diabetes) [20], there is scarce information about the relationship between exercise BP and CV function in adolescents. A cross-sectional analysis from the European Youth Heart Study found that adiposity (i.e. BMI) and insulin sensitivity (HOMR-IR) were independently (of each other) associated with exercise systolic BP in children (8-to-10-year-olds) and adolescents (14-to-16-year-olds) [21]. Nonetheless, the current study is the first to identify independent relationships between submaximal exercise BP and CV structure in a large, non-selected and apparently healthy adolescent population.

Whilst EEBP in middle-to-older-age may signal existing or underlying CV abnormalities, it is well-known that CV disease originates in early life. Raised BP ‘tracks’ from childhood/adolescence into adulthood [22,23], and, elevated BP in youth is a risk factor for development of hypertension and associated CVD in adulthood. One prior study of 274 healthy children aged 6 to 15 years stratified by tertiles of BP demonstrated a stronger association between exercise systolic BP and LV mass compared to baseline resting systolic BP [24]. Grontved et al. also found that systolic BP measured during cycle ergometry in young boys and girls (mean age 9.8 years) independently (of resting BP) predicted systolic BP six-years later during adolescence [25]. The current study is consistent with these findings and adds to them by showing that associations between post-exercise systolic BP and CV structure are not fully explained by DEXA-assessed body composition (Model 6, Table 2). Nonetheless, associations between exercise BP and cardiac structure (LV mass in particular) were heavily attenuated (although not abolished) by inclusion of lean body mass in regression models, which is consistent with the observations of Daniels et al. [26], who noted that up to 75% of the variance in LV mass may be explained by lean body mass in children and adolescents.

Accurate identification of abnormal BP and/or hypertension-related CVD risk in childhood/adolescence is of great importance, because it may provide an opportunity to intervene, perhaps via lifestyle modification and prevent adult hypertension and CVD events. Moreover, exercise BP may be a useful screening tool, since our recent studies have highlighted the clinical usefulness of submaximal exercise BP in adult risk prediction, because EEBP reveals the presence of underlying [4], or ‘masked’ hypertension [3] (and therefore underlying CV risk) that would go unnoticed by standard (resting) screening methods. Although majority of associations between post-exercise or recovery-exercise BP and CV structural variables (excluding aortic stiffness) were not independent of resting office BP as a continuous variable, in the absence of elevated resting office BP there is no clinical indication to further assess CV risk. Hence, the value of exercise BP is that it may reveal CV risk irrespective of office BP status. Exercise BP may also prove particularly useful as a screening tool in adolescents with other markers of CV risk, such as a significant family history of hypertension or CV disease. Although follow-up of the current cohort is required to confirm future CV risk associated with adolescent post-exercise systolic BP, our results suggest that during adolescence, it is possible to identify potentially increased hypertension-related CV risk from the BP response to a submaximal exercise test, irrespective of apparently normal office BP status.

### 4.1 Limitations

The step-test performed in this study did not allow for physiological measures to be performed at standardized workload during exercise, and therefore the intensity of effort at which time BP was measured may have differed across the cohort. Nonetheless, based on the post-exercise heart rate the intensity typically corresponded to ~55% of maximum, and BP measurement always occurred within the first 30 s following the step-test. Direct measures of aerobic capacity (peak VO_2_) were not available and it is possible that this may be related to the exercise BP response, as has been demonstrated previously [5,27]. Further studies should aim to quantify the relationship of aerobic capacity with exercise BP and CV risk in adolescents. Although no appreciable differences in associations between post-exercise and recovery BP with CVD markers were observed between the complete case analysis and the imputed data set, the complete case analysis presented assumes data to be missing at random. Moreover, this study was cross-sectional in nature and therefore causal pathways and future CV risk associated with post-exercise and recovery-exercise BP cannot be ascertained.

## 5 Conclusion

Systolic BP measured before and following a submaximal exercise test was associated with CV structure independently of body composition and hypertension status in a large cohort of adolescents. Measurement of submaximal exercise BP in youth may have utility for hypertension-related risk screening, and CV health in later life.

The following are the supplementary data related to this article.

Supplementary Table 1 Cardiovascular structure by sex. Supplementary Table 1

Supplementary Table 2 Pre-exercise and delta systolic BP and cardiovascular structure (sex-pooled analyses). Supplementary Table 2

## Disclosures

DAL has received support from Roche Diagnostics and Medtronic for biomarker research unrelated to the research presented here. All other authors report no conflicts of interest.
