# Supplementary material for: Submaximal exercise blood pressure and cardiovascular structure in adolescence
Source: Int J Cardiol. 2019 Jan 15;275:152–7. doi: 10.1016/j.ijcard.2018.10.060 (PMC6282652; doi:10.1016/j.ijcard.2018.10.060)
Supplement: Supplementary Table 1 — Cardiovascular structure by sex. [file mmc1.docx]

**Supplementary table 1.** Cardiovascular structure by sex.

|  | **Mean ± SD or N (%) in all participants** | | **Mean ± SD or N (%) in Females** | **Mean ± SD or N (%) in Males** | ***P* value** | **Effect size*** |
| --- | --- | --- | --- | --- | --- | --- |
| LV mass, g (n=2102) | | 123.82 ± 33.11 | 107.38 ± 22.94 | 143.99 ± 32.53 | <0.001 | 0.302 |
| LV mass index, g/m^2.7^ (n=2067) | | 28.76 ± 6.20 | 27.78 ± 5.72 | 29.96 ± 6.56 | <0.001 | 0.030 |
| LA size, cm (n=1903) | | 3.18 ± 0.41 | 3.07 ± 0.38 | 3.30 ± 0.41 | <0.001 | 0.081 |
| LV volume, ml (n=2100) | | 94.08 ± 21.54 | 84.67 ± 16.09 | 105.64 ± 21.77 | <0.001 | 0.235 |
| LV internal diameter, cm (n=2100) | | 4.51 ± 0.44 | 4.32 ± 0.35 | 4.74 ± 0.42 | <0.001 | 0.231 |
| Interventricular septum, cm (n=2102) | | 0.82 ± 0.13 | 0.79 ± 0.12 | 0.87 ± 0.13 | <0.001 | 0.093 |
| Posterior wall thickness, cm (n=2102) | | 0.87 ± 0.12 | 0.82 ± 0.11 | 0.92 ± 0.11 | <0.001 | 0.163 |
| Relative wall thickness (n=2099) | | 0.39 ± 0.06 | 0.38 ± 0.06 | 0.39 ± 0.06 | 0.009 | 0.003 |
| Aortic PWV, m/s (n=3582) | | 5.74 ± 0.69 | 5.52 ± 0.61 | 6.01 ± 0.68 | <0.001 | 0.125 |
| Carotid IMT, cm (n=3721) | | 0.48 ± 0.04 | 0.47 ±0.05 | 0.49 ± 0.05 | <0.001 | 0.023 |

Data are mean ± SD. All measures reported at end-diastole. LV, left ventricular; PWV, pulse wave velocity; IMT, intima-media thickness. P values relate to comparison of males vs. females and were calculated using a *t*-test. *Partial eta squared.
