# Supplementary material for: Submaximal exercise blood pressure and cardiovascular structure in adolescence
Source: Int J Cardiol. 2019 Jan 15;275:152–7. doi: 10.1016/j.ijcard.2018.10.060 (PMC6282652; doi:10.1016/j.ijcard.2018.10.060)
Supplement: Supplementary Table 2 — Pre-exercise and delta systolic BP and cardiovascular structure (sex-pooled analyses). [file mmc2.docx]

**Supplementary table 2.** Pre-exercise and delta systolic BP and cardiovascular structure (sex-pooled analyses).

| **B. Post-exercise - Pre-exercise (delta) systolic BP** | | | | | | | |
| --- | --- | --- | --- | --- | --- | --- | --- |
|  | **Model 1** | **Model 2** | **Model 3** | **Model 4** | **Model 5** | **Model 6** |  |
|  | **β (95% CI) per 5 mmHg SBP** | **β (95% CI) per 5 mmHg SBP** | **β (95% CI) per 5 mmHg SBP** | **β (95% CI) per 5 mmHg SBP** | **β (95% CI) per 5 mmHg SBP** | **β (95% CI) per 5 mmHg SBP** |  |
| **LV Mass, g**  **(n=1,768)** | -1.039 (-1.700, -0.378) * | -1.039 (-1.700, -0.378) * | -1.501 (-2.177, -0.824) * | 0.805 (0.335, 1.275) * | -0.031 (-0.478, 0.404) | -0.041 (-0.482, 0.400) |  |
| **LVMI, g/m^2.7^**  **(n=1,756)** | 0.062 (-0.062, 0.187) | 0.062 (-0.062, 0.187) | -0.122 (-0.45, 0.001) | 0.204 (0.082, 0.325) * | 0.006 (-0.110, 0.121) | 0.005 (-0.110, 0.120) |  |
| **LA size, cm**  **(n=1,603)** | 0.000 (-0.008, 0.009) | 0.000 (-0.008, 0.009) | -0.012 (-0.020, -0.003) * | 0.013 (0.005, 0.021) * | -0.001 (-0.008, 0.007) | -0.001 (-0.008, 0.007) |  |
| **RWT**  **(n=1,768)** | 0.000 (-0.001, 0.001) | 0.000 (-0.001, 0.002) | 0.000 (-0.001, 0.001) | 0.001 (-0.001, 0.002) | 0.000 (-0.001, 0.002) | 0.000 (-0.001, 0.002) |  |
| **Aortic PWV, m/s**  **(n=2,965)** | -0.011 (-0.021, -0.000) * | -0.012 (-0.023, -0.001) * | -0.004 (-0.015, 0.007) | 0.008 (-0.003, 0.018) | 0.009 (-0.002, 0.020) | 0.008 (-0.003, 0.018) |  |
| **cIMT, mm**  **(n=3,687)** | -0.001 (-0.002, 0.000) * | -0.001 (-0.002, 0.000) | 0.001 (-0.001, 0.000) | 0.000 (0.000, 0.001) | 0.000 (0.000, 0.001) | 0.001 (0.000, 0.001) |  |

Results presented as unit change (β) in outcome per 5 mmHg. LV, left-ventricular; LA, left-atrial; RWT, relative wall thickness; cIMT, carotid intima media thickness; PWV, pulse wave velocity. * indicates confidence intervals do not cross zero. **Model 1** - univariable; **Model 2** - adjusted for age (years); **Model 3** - model 2 plus adjustment for total body fat mass (kg); **Model 4** - model 2 plus adjustment for total lean mass (kg); **Model 5** - model 2 plus adjustment for total body fat mass (kg) and total lean mass (kg); **Model 6** - model 5 plus adjustment for hypertension status (resting SBP and/or DBP ≥ 140/90 mmHg - yes/no);
